# The complete mitochondrial genome of the *Riparia riparia* (Passeriformes: Hirundinidae)

**DOI:** 10.1080/23802359.2022.2090296

**Published:** 2022-06-28

**Authors:** Jie Huang, Baodong Yuan, Huaming Zhong, Xianmeng Shi, Bo Yang, Jianjun Peng, Chengzhong Yang

**Affiliations:** a College of Biology and Food, Shangqiu Normal University, Shangqiu, China; b China Conservation and Research Centre for the Giant Panda, Dujiangyan, China; c School of Life and Health Sciences, Hunan University of Science and Technology, Xiangtan, China; d College of Life Sciences, Chongqing Key Laboratory of Animal Biology, Chongqing Normal University, Chongqing, China

**Keywords:** *Riparia riparia*, mitochondrial genome, phylogenetic analysis

## Abstract

The Sand Martin (*Riparia riparia*) belongs to Hirundinidae. In this study, the complete mitochondrial genome of *R. riparia* was sequenced and characterized. The genome was 17,963 bases in length (GenBank accession no. OK537984) including 13 protein-coding genes, two ribosomal RNA (rRNA) genes, 22 transfer RNA (tRNA) genes, and two control regions. The overall base composition of *R. riparia* mitogenome was 30.5% for A, 31.8% for C, 14.5% for G, and 23.2% for T. Phylogenetic analysis revealed that *R. riparia* was genetically closest to the species of genus *Tachycineta*. *R. riparia* mitogenome could contribute to our understanding of the phylogeny and evolution of this species.

In this study, we focus on the Sand Martin (*Riparia riparia* Linnaeus 1758), a threatened migratory bird (BirdLife International 2017), which is mostly distributed in the plains in the western, northern, and northeastern parts of Eurasia (Meklenburtsev 1954). The Sand Martin’s natural nesting habitats are sandy banks of rivers, streams and lakes (Cramp 1988). Goroshko (1993) also indicates that *R. riparia* nests near rivers with floodplain vegetation and a rich grass cover and shrub thickets. Although the behavior ecology (Zhou et al. 2004; Ye et al. 2016; Saldanha et al. 2019) or genetic structure research based on ND2 (An et al. 2019) has been carried out in recent years, the complete mitochondrial data of *R. riparia* were still lacking.

In this study, we sequenced the complete mitochondrial genome of *R. riparia* (GenBank accession no. OK537984). The muscle sample was obtained from a wild *R. riparia* that died of natural causes in national wetland park of the Yellow River original course, Henan Province, China (E115°26.22′, N34°66.85′). The specimen was deposited at the College of Biology and Food, Shangqiu Normal University (Huaming Zhong; monzhm@126.com) under the voucher number SQSW002. Genomic DNA was extracted from the sample using a DNeasy Blood and Tissue kit (Qiagen, Valencia, CA). Primers were designed according to the mitochondrial genomic sequences of closely related species. The complete mitochondrial genome sequence of *R. riparia* was amplified and sequenced by these primers using Sanger sequencing technology.

The complete mitochondrial genome of the *R. riparia* was 17,963 bp in length, contains 22 transfer RNA (tRNA) genes, 13 typical protein-coding genes, two ribosomal RNA (rRNA) genes (12S rRNA and 16SrRNA), and two control region (CR) (D-loop1 and D-loop2). This gene arrangement is similar to that found in other passerines (Shuo et al. 2016; Huang et al. 2019). The base composition of mtDNA is 30.5% A, 23.2% T, 31.8% C, and 14.5% G, so the percentage of A + T (53.7%) was slightly higher than G + C (46.3%). In 13 PCGs, the shortest one was ATP8 gene (168 bp) and the longest one was the ND5 gene (1818 bp). The usage of the start codon was mainly ATG in the most of mitochondrial protein coding genes besides the COI gene employing the GTG and the ND3 gene employing the ATA; the usage of the stop one was either complete (TAA, TAG, AGA, and AGG) or incomplete (T– –). The two CRs were 1081 bp and 1310 bp long, and they had a continuous region (1041 bp) of near identical sequence (>99% sequence identity). The CR1 had no repeat units detected, while the CR2 had a microsatellite consisting of a series of complex cytosine–adenine repeats.

A phylogenetic tree was constructed based on 13 protein-coding gene sequences from *R. riparia* and 22 other Hirundinidae species using the maximum-likelihood (ML) method of MEGA version 7.0 with 1000 bootstrap replicates (Kumar et al. 2016). The ML tree was based on the Kimura 2-parameter model of nucleotide substitution. The results indicated that the clade of *R. riparia* was the sister lineage to the clade formed by the species of genus *Tachycineta* (Figure 1). The complete mitogenomes of *R. riparia* will provide a better understanding of the phylogeny and evolutionary analysis of Hirundinidae.

**Figure 1.**
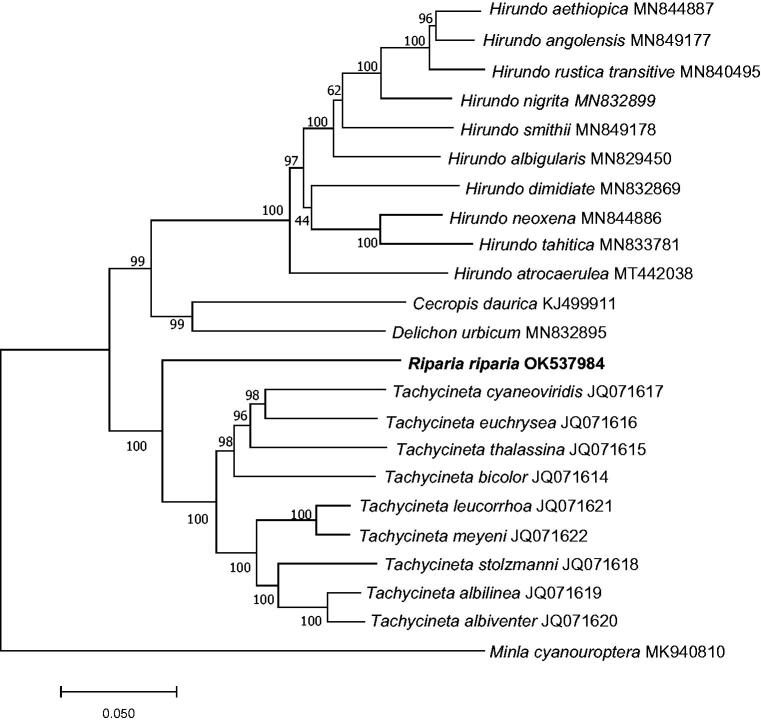
Maximum-likelihood (ML) tree based on 13 protein-coding gene sequences of 22 species of Hirundinidae, with *Minla cyanouroptera* as the outgroup. The numbers on the branch lengths are bootstrap values. The species names are followed by their GenBank accession numbers.

## Data Availability

The genome sequence data that support the findings of this study are openly available in GenBank of NCBI at https://www.ncbi.nlm.nih.gov/ under the accession no. OK537984. The associated BioProject and BioSample numbers are PRJNA810090 and SAMN25995379, respectively.
